# Syntheses, Structures and Antimicrobial Activities of bis(Imino)acenaphthene (BIAN) Imidazolium Salts

**DOI:** 10.3390/molecules16043168

**Published:** 2011-04-15

**Authors:** Rachel R. Butorac, Salem S. Al-Deyab, Alan H. Cowley

**Affiliations:** 1 Department of Chemistry and Biochemistry, University of Texas at Austin, 1 University Station, A5300, Austin, TX 78712, USA; 2 Chemistry Department, College of Science, King Saud University, P.O. Box 2455, Riyadh 11451, Saudi Arabia

**Keywords:** imidazolium chloride, BIAN, synthesis, X-ray structure, antimicrobial activity

## Abstract

The syntheses of four new bis(imino)acenaphthene (BIAN) imidazolium chlorides are reported, three of which have been structurally characterized. The synthesis of a new, structurally authenticated BIAN ligand is also described. We report the results of the use of these BIAN imidazolium salts as antimicrobials against the pathogens *S. aureus*, *B. subtilis*, *E. coli* and *P. aeruginosa*. The antimicrobial efficacies were particularly high for the N-(2,6-diisopropylphenyl)- and N-(mesityl)- substituted BIAN imidazolium salts (MIC values < 0.6 μg/mL).

## 1. Introduction

It has been known for over a century that quaternary ammonium compounds (QACs) possess antibacterial properties [1]. More recently [2] it has been recognized that an important aspect of the initial attack of a bacterial cell by the QAC involves an electrostatic interaction with the phospholipid bilayer. In turn, this interaction can compromise the integrity of the cytoplasmic membrane, thus causing respiratory inhibition and intracellular coagulation [3]. Given the electrostatic nature of the initial phase of the biocidal process, interest has been generated in the synthesis and antimicrobial properties of other cationic nitrogen containing molecules and polymers [4]. Generally speaking, small molecule antimicrobial agents are excellent in terms of cell wall diffusion capability [3].

The present work is focused on the antimicrobial behavior of imidazolium salts. Much of the early work in this area was descrbed by Pernak *et al.* [5]. For example, these authors noted a quantitative correlation between the minimum inhibitory concentration (MIC value) [6], the critical micelle concentration and hydrophobicity in the case of 3-alkylthiomethyl-1-ethylimidazolium chlorides. In subsequent work [7] with 3- alkoxymethyl-1-methylimidazolium salts, it was shown that the compounds with 10, 11, 12, and 14 carbon atoms associated with the alkoxy group exhibited high antimicrobial activities. The MIC values ranged from 1.13–11.9 μg/mL for the C_10_H_21_-substituted salt to 0.69–46 μg/mL for the C_12_H_25_ analogue (for reference, the MIC value for AgNO_3_ is between 0.1 and 0.05 μg/mL) [8]. The foregoing results were independent of the type of anion employed.

**Figure 1:**
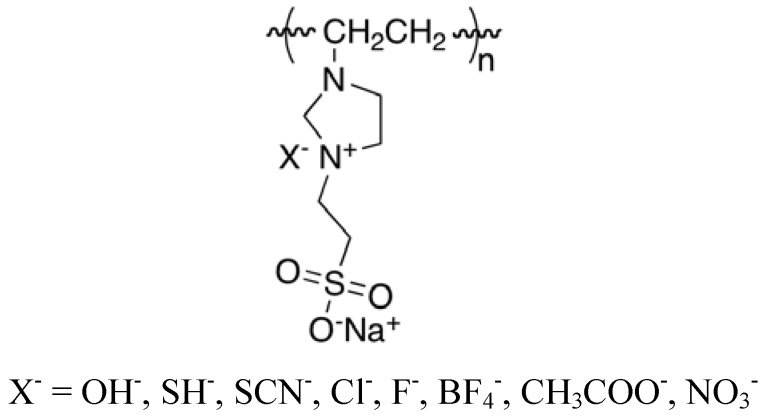
Structure of poly[1-vinyl-3-(2-sulfoethylimidazolium betaine)].

More recently, Lee *et al.* [9] synthesized and studied two comparable series of antimicrobial compounds, namely 1-alkyl-3-hydroxyethyl-3-methylimidazolium chlorides and 1-alkyl-3-methyl-imidazolium halides. The chloride and bromide salts of the latter class of compounds were found to be the most effective antibacterial agents and the preferred alkyl chain lengths fell in the range C_12_ – C_16_. For example, the MIC values for the C_14_H_29_-substituted chloride and bromide salts were 4 μg/mL against *E. coli*, *S. aureus* and *B. subtilis*. In contrast to the foregoing, the antimicrobial activity of the functional polymer poly[1-vinyl-3-(2-sulfoethylimidazolium betaine)] (Figure 1) was found to be sensitive to the nature of the counteranion [10]. The anion dependence in this case is attributable to the anion-dependent changes in the alignment of the polymer chains.

Çetinkaya *et al.* [11] have examined the antimicrobial activities of several saturated analogues of imidazolium salts, namely 1,3-diorganylimidazolidinium salts. These authors discovered that, while there was a dependence of activity on the length of the *n*-alkyl chains, the attachment of mesityl or mesitylmethyl substituents to the N atoms resulted in the most significant increases of activity towards both Gram-positive and Gram-negative bacteria. Interestingly, however, in contrast to the work of Pernak *et al.* [2] the activities of these derivatives were found to depend on the identity of the counter anion. For example, the MIC value for the mesitylmethyl chloride salt was 3.12 μg/mL.

In the present manuscript, we describe a new class of potential antimicrobial agent, namely bis(imino)acenaphthene (BIAN) imidazolium salts. The BIAN class of ligand, which features the fusion of a naphthalene moiety to a diimine, has a number of distinctive properties, one of which is facile redox behavior. To the best of our knowledge, there is only one reference [12] to the use of BIAN-supported antimicrobial compounds, namely the neutral compounds bis[N-(2,6-diisopropylphenyl)imino]acenaphthene (Pr-BIAN), [Co(Pr-BIAN)Cl_2_], [Co(OAc)_2_(Pr-BIAN)_2_][ClO_4_], [Ni(Pr-BIAN)(NO_3_)_2_] and [Ni(Pr-BIAN)_2_][ClO_4_]_2_. The most active compounds against *S. aureus*, *E. coli*, and *C. albicans* were found to be [Co(Pr-BIAN)Cl_2_], [Co(OAc)_2_(Pr-BIAN)_2_] and Pr-BIAN. The MIC values for these compounds fell within the range 1.7–2.6 mg/mL. Interestingly, bis[N-(*p*-tolylphenyl)imino]acenaphthene, the other BIAN ligand that was tested, was found to be inactive, thus suggesting that the antimicrobial activity is dependent upon the steric bulk of the N-substituents. In a previous publication we have discussed the antimicrobial behavior of BIAN-N-heterocyclic carbene complexes of silver and gold [13].

## 2. Results and Discussion

### 2.1. Syntheses of **L**, **2**, **3**, **4** and **5**

**Figure 2:**
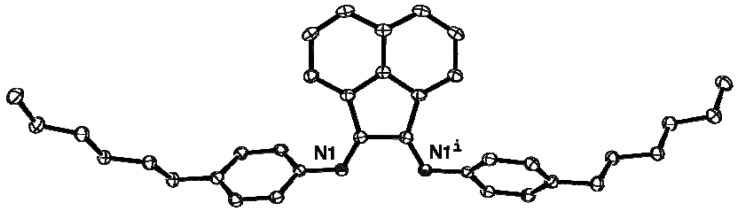
ORTEP diagram of **L** with 50% probability thermal ellipsoids. Hydrogen atoms are omitted for clarity. **L **lies on a two-fold axis (-x, y, 3/2-z) and as a result, N1^i^ is equivalent to N1.

The new 4-*n*-hexyl(BIAN) ligand (**L**) shown in Figure 2 was prepared by treatment of acenaphthenequinone with 4-*n*-hexylaniline. Ligand **L**, along with the known ligands dipp(BIAN), mes(BIAN), *p*-MeO(BIAN) and *p*-F(BIAN) were converted into the corresponding imidazolium salts by treatment with methoxy(methyl)chloride. Details of these syntheses are provided in the Experimental Section.

### 2.2. X-ray Crystal Structures of **L**, **2**, **4** and **5**

**Figure 3:**
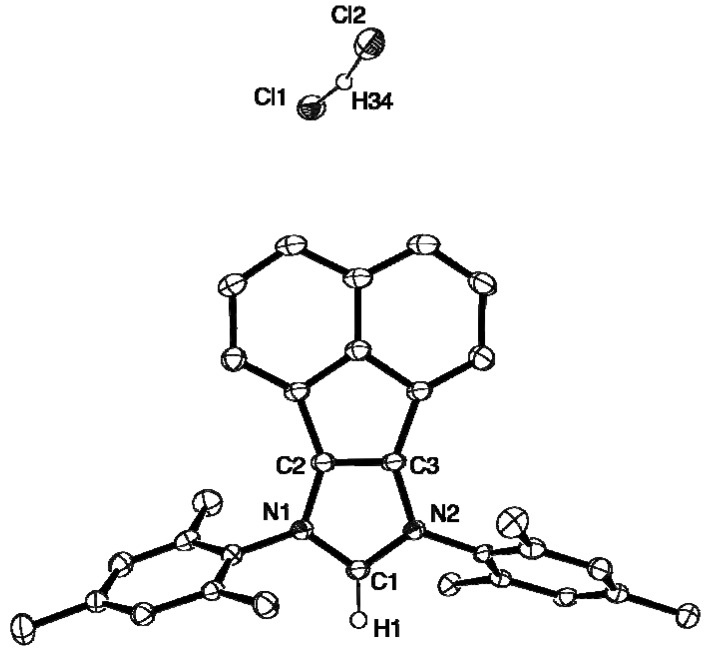
ORTEP diagram of **2** with 50% probability thermal ellipsoids. Hydrogen atoms except H1 and H34 are omitted for clarity.

**Figure 4:**
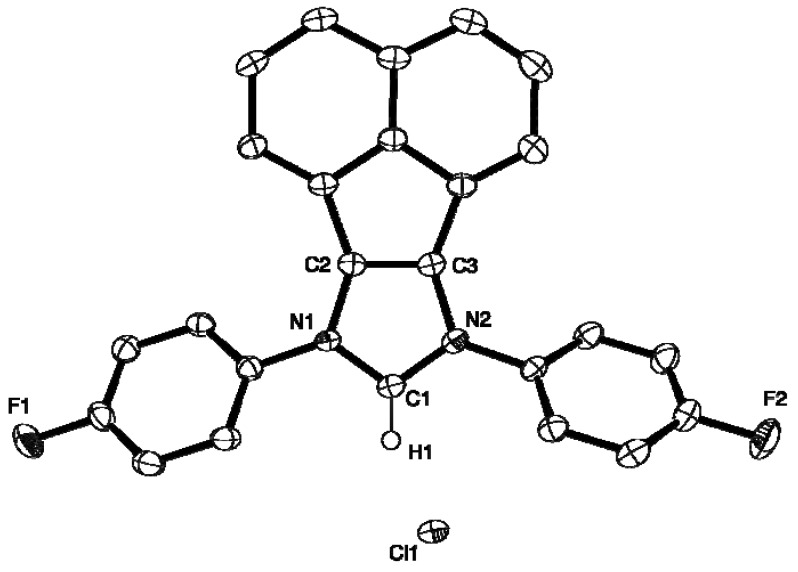
ORTEP diagram of **4** with 50% probability thermal ellipsoids. Hydrogen atoms except H1 are omitted for clarity.

**Figure 5:**
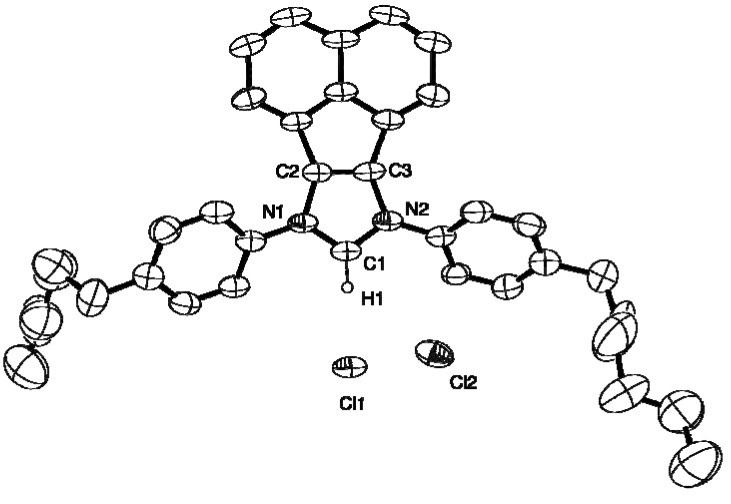
ORTEP diagram of **5** with 40% probability thermal ellipsoids. Hydrogen atoms except H1 are omitted for clarity. The hydrogen atom of the anion [HCl_2_]^-^ was not detected in the difference map.

The molecular structures of **L**, **2**, **4** and **5** are presented as ORTEP diagrams in Figure 2, Figure 3, Figure 4, Figure 5. A summary of X-ray data collection details appears in Table 1 and a selection of pertinent metrical parameters for **2**, **4** and **5 ** is available in Table 2. Unfortunately, despite several attempts, it was not possible to grow suitable crystals of the *p*-MeO-substituted analogue **3**. As expected, given the rigidity of the BIAN framework, the bond distances and angles for **2**, **4** and **5** (Table 2) are very similar to each other and, in turn, to those already reported for IPr(BIAN) (**1**) [14]. There are, however, differences in the counter-anions in the sense that **2** and **5** crystallize with a hydrogen dichloride anion rather than a chloride anion. Although somewhat rare, salts of this anion have been prepared by e.g. the reaction of crown ethers with hydrogen chloride, water and 18-crown-6 in toluene and the reaction of an alkali-metal chloride with HCl in aqueous media [15,16].

**Table 1:** Selected crystal data, data collection and refinement parameters for compounds **L**, **2**, **4** and **5**.

	L	2	4	5
Formula	C_36_H_40_N_2_	C_33_H_35_Cl_3_N_2_O	C_26_H_17_Cl_3_F_2_N_2_	C_37_H_42_Cl_2_N_2_
Formula weight	500.70	581.98	501.77	585.63
Crystal system	Monoclinic	Monoclinic	Monoclinic	Monoclinic
Space group	C2/c	P2_1_/c	P2_1_/c	P2_1_/c
*a*/Å	31.987(3)	12.7377(12)	10.104(3)	17.047(4)
*b*/Å	9.0070(9)	14.4165(13)	24.514(7)	12.711(3)
*c*/Å	10.3906(10)	16.2893(14)	9.243(2)	14.823(4)
*α*/°	90.0	90.0	90.0	90.0
*β*/°	105.809(4)	91.280(3)	94.103(3)	90.142(5)
*γ*/°	90.0	90.0	90.0	90.0
Z	4	4	4	4
*D*_c_/g cm^-3^	1.155	1.293	1.459	1.211
F(000)	1080	1224	1024	1248
Crystal size/nm	0.24 × 0.18 × 0.06	0.16 × 0.14 × 0.11	0.26 × 0.16 × 0.14	0.23 × 0.20 × 0.09
*Θ* range/°	3.00–27.48	3.00–25.00	2.02–27.50	2.99–25.00
Collected reflections	29622	24514	25427	19946
Independent reflns	3304	5172	5213	5644
R_int_	0.0602	0.1143	0.0583	0.0808
*R*_1_ [*I*>2σ(*I*)]	0.0502	0.0529	0.0567	0.0878
w*R*_2_ (all data)	0.1226	0.1375	0.1785	0.2421

**Table 2:** Selected bond distances (Å) and angles (°) for compounds **2**, **4** and **5**.

Bond distances/Å	**2**	**4**	**5**
C(1)-N(1)	1.351(3)	1.347(3)	1.339(5)
C(1)-N(2)	1.346(3)	1.345(3)	1.341(5)
C(2)-C(3)	1.368(4)	1.371(3)	1.350(6)
C(2)-N(1)	1.375(3)	1.383(3)	1.386(5)
C(3)-N(2)	1.390(3)	1.383(3)	1.385(5)
Bond angles/°	**2**	**4**	**5**
N(1)-C(1)-N(2)	109.5(2)	109.08(18)	110.0(4)
N(1)-C(2)-C(3)	107.8(2)	107.45(19)	107.2(4)
C(2)-C(3)-N(2)	107.3(2)	107.10(19)	108.3(4)
C(1)-N(1)-C(2)	107.8(2)	108.03(18)	107.5(4)
C(1)-N(2)-C(3)	107.6(2)	108.33(17)	106.9(4)

### 2.3. Antimicrobial Testing of Compounds **1-5**

In the present work, we report the results of the use of BIAN imidazolium salts as antimicrobials. As mentioned in the Introduction, the previous use of BIAN ligands was focused on neutral species and N-heterocyclic carbene derivatives thereof [13]. Since the initial phase of the attack of bacterial cell walls by antimicrobials is believed to be electrostatic in nature [3], it was thought that the use of cationic BIAN species might prove to be advantageous.

It is clear from the work of El-Ayaan *et al. * [12] and Çetinkaya *et al.* [11] that the nature of the nitrogen substituents on the cation can play an important role in terms of antimicrobial activity. Accordingly, we chose to compare the activities of IPr(BIAN) imidazolium chloride **1** and mes(BIAN) imidazolium chloride **2**. The *p*-MeO(BIAN) and *p*-F(BIAN) imidazolium chlorides **3** and **4** were selected in order to explore the sensitivity of antimicrobial activity to the presence of electron donating and electron withdrawing groups, respectively on the nitrogen substituents. Finally, the 4-*n*-hexyl(BIAN) imidazolium chloride **5** was chosen in order to probe the possible influence of the presence of long alkyl chains on the para positions of the N-aryl groups.

**Table 3:** Minimum inhibitory concentrations (MIC) for imidazolium salts **1**-**5 **in μg/mL.

	Microbial spp.			
Compound	S. aureus	B. subtilis	E. coli	P. aeruginosa
**1**	<0.6	<0.6	39.06	39.06
**2**	<0.6	<0.6	78.12	39.06
**3**	1.22	4.88	312.55	>312.55
**4**	19.53	39.06	>312.55	>312.55
**5**	9.76	9.76	>312.55	>312.55

Based on the MIC values presented in Table 3, the relative activities of the five imidazolium salts against the four pathogens tested can be summarized a follows:

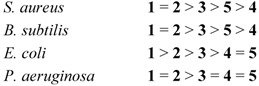


The most obvious features of the antimicrobial efficacy data presented in Table 3 are the high activities of dipp(BIAN) imidazolium chloride **1** and mes(BIAN) imidazolium chloride **2** toward the Gram-positive bacteria *S. aureus* and *B. subtilis*. In both cases, the MIC values are < 0.6 μg/mL. It is also noteworthy that, while the MIC values are larger for the Gram-negative bacteria *E. coli* and *P. aeroginosa*, **1** and **2** are still the most active imidazolium salts. The lower bactericidal activity toward the Gram-negative bacteria is presumably a consequence of their more complex, less porous cell wall structures [3]. There is also an interesting parallel with the results of Çetinkaya *et al. * [11] in the sense that these authors noted that the attachment of mesityl or methylmesityl groups to both nitrogen atoms of imidazolidinium salts resulted in high activities against both Gram-positive and Gram-negative bacteria.

The importance of a positive charge on BIAN-supported antimicrobials is highlighted by the report that the MIC values for the neutral compound Pr-BIAN against *S. aureus*, *E. coli* and *C. albicans* are 2.1, 2.0 and 2.3 mg/mL, respectively [12]. The fact that the MIC values for *p*-MeO(BIAN) **3** against *S. aureus* and *B. subtilis* are less than those for *p*-F(BIAN) **4** may be due to the stabilization of **3 **by electron donation from the methoxy substituent.

## 3. Experimental

### 3.1. General

All glassware was oven-dried before use. All reagents were obtained commercially and used as received. The neutral ligands mes(BIAN) [17], *p*-F(BIAN) [18], and *p*-MeO(BIAN) [19] and the salt IPr(BIAN) imidazolium chloride (**1**) [14] were prepared according to literature procedures. 

### 3.2. Physical measurements

Low-resolution CI mass spectra were obtained on a Thermo Scientific TSQ Quantum GC mass spectrometer and high-resolution CI mass spectra were recorded on a magnetic sector Waters Autospec Ultima instrument. ^1^H- and ^13^C{^1^H}-NMR spectra were recorded at 295 K on a Varian INOVA 500 spectrometer (^1^H, 500 MHz; ^13^C, 125 MHz) immediately following sample preparation. Deuterated chloroform, deuterated methylene chloride and deuterated dimethyl sulfoxide were obtained from Cambridge Isotopes and stored over 4 Å molecular sieves prior to use. The ^1^H- and ^13^C{^1^H} chemical shift values are reported in parts per million (ppm) relative to SiMe_4_ (δ 0.00), using the solvent resonance as the internal standard.

### 3.3. Preparations

4*-n-hexyl(BIAN) ligand *(**L**). Acenaphthenequinone (0.2 g, 1.098 mmol) and 4-*n*-hexylaniline (0.47 mL, 2.41 mmol) were added to a 100 mL round bottom flask. Glacial acetic acid (20 mL) was added slowly. The yellow-orange suspension was refluxed for 2 h and then cooled to room temperature. The mixture was filtered and washed with glacial acetic acid (2 × 15 mL) and finally diethyl ether (3 × 20 mL). The resulting orange solid was added to a 100 mL round bottom flask and refluxed for 2 h with 15 mL of a K_2_CO_3_ solution (10 g of K_2_CO_3_ in 15 mL H_2_O). The mixture was cooled to room temperature and the aqueous layer was extracted with methylene chloride (3 × 20 mL). The organic layer was washed with water (3 × 15 mL), following which the resulting solution was dried over MgSO_4_ and evaporated to dryness to give a yellow-orange crystalline analytically pure powder (0.398 g, 72.4%). Crystals of **L **suitable for X-ray diffraction experiments were obtained from a saturated THF/hexanes solution that had been stored at ambient temperature for 7 days. MS (CI^+^, CH_4_): *m/z* 501 [M+H]^+^ (+ H); HRMS (CI^+^, CH_4_): calcd for C_36_H_40_N_2_ *m/z *500.3191; found, 500.3171; ^1^H-NMR (CDCl_3_): *δ* 0.90 (t, 6H, -CH_3_), 1.32–1.40 (m, 12H, -CH_2_-CH_2_CH_2_-), 1.69 (quin, 4H, -CH_2_), 2.67 (t, 4H, -CH_2_), 6.86 (d, 2H, Naph-H), 7.02 (d, 4H, Ar-H), 7.24 (d, 4H, Ar-H), 7.33 (t, 2H, Naph-H), 7.85 (d, 2H, Naph-H); ^13^C-NMR (CDCl_3_): *δ* 14.07, 22.63, 28.95, 31.47, 31.72, 35.50, 118.13, 123.81, 127.51, 128.70, 128.73, 129.23, 131.18, 139.01, 141.65, 149.42, 161.29; m.p.: 136–138 °C.

*Mes(BIAN) imidazolium chloride *(**2**). Mes(BIAN) (0.200 g, 0.48 mmol) and methoxy(methyl)chloride (3.22 g, 40.0 mmol) were added to an argon-flushed thick-walled reaction vessel. The vessel was sealed and the reaction mixture was stirred at 100 °C for 16 h, during which time the appearance of the mixture changed from a murky black suspension to a clear dark-red solution. Cooling of the reaction mixture to ambient temperature and the subsequent addition of 10 mL of diethyl ether resulted in the formation of a yellow precipitate. The resulting solid was filtered off and washed with 50 mL of diethyl ether and dried *in vacuo *to afford **2** as an analytically pure yellow powder (0.186 g, 83.4%). Crystals of **2** suitable for X-ray diffraction experiments were isolated from the reaction mixture and stored in anhydrous diethyl ether. MS (CI^+^, CH_4_): *m/z* 429 [M+H]^+ ^(-Cl); HRMS (CI^+^, CH_4_): calcd for C_31_H_29_N_2_ *m/z *429.2331; found, 429.2325; ^1^H-NMR (CDCl_3_): *δ* 2.30 (s, 12H, Ar-CH_3_), 2.40 (s, 6H, Ar-CH_3_), 7.12 (s, 4H, Ar-H), 7.27 (d, 2H, Naph-H), 7.54 (d of d, 2H, Naph-H), 7.95 (d, 2H, Naph-H), 11.37 (s, 1H, C-H); ^13^C-NMR (CDCl_3_): *δ* 17.89, 21.26, 122.81, 123.50, 128.14, 129.74, 129.83, 130.09, 130.21, 134.13, 136.58, 141.37, 142.52; m.p. (decomp): 256–259 °C.

*p-MeO(BIAN) imidazolium chloride *(**3**). Compound **3** was prepared in a similar fashion to that described for **2** starting with *p*-MeO(BIAN) (0.216 g, 0.55 mmol) and methoxy(methyl)chloride (3.22 g, 40.0 mmol). The resulting olive green precipitate was filtered off and washed with 50 mL of diethyl ether and dried *in vacuo *to afford **3** as an analytically pure olive green powder (0.220 g, 90.5%). MS (CI^+^, CH_4_): *m/z* 405 [M+H]^+ ^(-Cl); HRMS (CI^+^, CH_4_): calcd for C_27_H_21_N_2_O_2_ *m/z *405.1603; found, 405.1600; ^1^H-NMR [(CD_3_)_2_SO]: *δ* 3.93 (s, 6H, -CH_3_), 7.36 (d, 4H, Ar-H), 7.71 (d of d, 2H, Naph-H), 7.84 (d, 2H, Naph-H), 8.04 (d, 4H, Ar-H), 8.15 (d, 2H, Naph-H), 10.11 (s, 1H, C-H); ^13^C-NMR [(CD_3_)_2_SO]: *δ* 55.83, 115.53, 122.86, 123.57, 125.22, 126.72, 128.27, 129.40, 129.50, 130.06, 135.05, 138.43, 160.70; m.p. (decomp): 193–195 °C.

*p-F(BIAN) imidazolium chloride (***4**). Compound **4** was prepared in a similar fashion to that described for **2** starting with *p*-F(BIAN) (0.211 g, 0.572 mmol) and methoxy(methyl)chloride (3.22 g, 40.0 mmol). The resulting yellow precipitate was filtered off and washed with 50 mL of diethyl ether and dried *in vacuo *to afford **4** as an analytically pure yellow powder (0.229 g, 95.8%). Crystals of **4** suitable for X-ray diffraction experiments were grown from a saturated CH_2_Cl_2_/hexanes solution stored at −40 °C. MS (CI^+^, CH_4_): *m/z* 381 [M+H]^+ ^(-Cl); HRMS (CI^+^, CH_4_): calcd for C_25_H_15_N_2_F_2_ *m/z *381.1203; found, 381.1195; ^1^H-NMR (CD_2_Cl_2_): *δ* 7.49 (d, 4H, Ar-H), 7.68 (t, 2H, Naph-H), 7.84 (d, 2H, Naph-H), 8.08 (d, 2H, Naph-H), 8.38 (d, 4H, Ar-H), 11.23 (s, 1H, C-H); ^13^C-NMR [(CD_3_)_2_SO]: *δ* 117.55, 117.74, 122.63, 123.93, 126.36, 126.43, 128.35, 129.43, 129.46, 130.34, 135.15, 139.31, 161.89, 163.87; m.p. (decomp): 274–276 °C.

4*-n-hexyl(BIAN) imidazolium chloride *(**5**). Compound **5** was prepared in a similar fashion to that described for **2** starting with *p*-*n*-hexyl(BIAN) (0.213 g, 0.425 mmol) and methoxy(methyl)chloride (3.22 g, 40.0 mmol). The resulting olive green/brown precipitate was filtered off and washed with 50 mL of diethyl ether and dried *in vacuo *to afford **5** as an analytically pure olive green powder (0.167 g, 71.7%). Crystals of **5** suitable for X-ray diffraction experiments were grown from a saturated CH_2_Cl_2_/hexanes solution stored at −40 °C. MS (CI^+^, CH_4_): *m/z* 513 [M+H]^+ ^(-Cl); HRMS (CI^+^, CH_4_): calcd for C_37_H_41_N_2_ *m/z *513.3270; found, 513.3260; ^1^H-NMR (CD_2_Cl_2_): *δ* 0.92 (t, 6H, -CH_3_), 1.34–1.44 (m, 12H, -CH_2_-CH_2_-CH_2_-), 1.72 (quint, 4H, -CH_2_), 2.77 (t, 4H, -CH_2_), 7.54 (d, 4H, Ar-H), 7.60 (d of d, 2H, Naph-H), 7.78 (d, 2H, Naph-H), 8.00 (d, 2H, Naph-H), 8.17 (d, 2H Ar-H), 11.16 (s, 1H, C-H); ^13^C-NMR (CD_2_Cl_2_): *δ* 14.19, 22.93, 29.34, 31.54, 32.00, 35.99, 123.55, 123.67, 124.01, 128.20, 130.18, 130.66, 130.69, 132.07, 135.78, 138.68, 146.81; m.p. (decomp): 218–221 °C.

### 3.4. X-ray crystallography

For compounds **L**, **2**, **4** and **5 **a crystal of suitable quality was covered with mineral oil and mounted on a nylon thread loop. The X-ray diffraction data were collected on either a Nonius Kappa CCD diffractometer equipped with an Oxford Cryostream low-temperature device cooled to 153 K or a Rigaku AFC-12 Saturn 724+ CCD diffractometer with a Rigaku XStream low temperature device cooled to 100 K. Both instruments were equipped with a graphite-monochromated Mo Kα radiation source (λ = 0.71073 Å). Corrections were applied for Lorentz and polarization effects. The structures were solved by direct methods and refined by full-matrix least-squares cycles on F^2^ using the Siemens SHELXTL PLUS 5.0 (PC) software package [20] and PLATON [21]. All non-hydrogen atoms were refined anisotropically, and hydrogen atoms were placed in fixed, calculated positions using a riding model. 

The crystallographic data for this paper have been deposited at the Cambridge Crystallographic Data Centre. These data can be obtained free of charge via www.ccdc.cam.ac.uk/conts/retrieving.html (or from the CCDC, 12 Union Road, Cambridge CB2 1EZ, UK; fax: +44 1223 336033; Email: deposit@ccdc.cam.ac.uk). The CCDC reference numbers for compounds **L**, **2**, **4** and **5** are 813271, 811058, 811059 and 811060, respectively.

### 3.5. Antimicrobial studies

Antimicrobial activities of compounds **1**-**5 **were determined using the Microtiter-Based Minimum Inhibitory Concentration (MIC) Test. *S. aureus *ATCC 6538, *B. subtilis *ATCC 19659, *E. coli *ATCC 11229 and *P. aeruginosa *ATCC 15442 were grown to approximetly 10^5^ CFU/mL in Mueller-Hinton broth. The stock solutions of compounds **1**-**5 **were prepared with DI water supplemented with dimethyl sulfoxide (DMSO). The concentrations of the tested compounds ranged from 0.03125% to 0.000006% using the Mueller-Hinton broth as the diluent, supplemented with DMSO to a final concentration of 2%. The plates were incubated at 36.0 ± 1 °C for 18–24 h. The MIC was taken to be the last well in the dilution series that did not exhibit growth as determined on the basis of turbidity.

## 4. Conclusions

In summary, four new bis(imino)acenaphthene (BIAN) imidazolium chlorides have been prepared and structurally authenticated. Along with the known N(2,6-diisopropylphenyl)imidazolium chloride, these salts have been tested for their antimicrobial activities against the Gram-positive bacteria *S. aureus* and *B. subtilis* and the Gram-negative bacteria *E. coli* and *P. aerginosa*. The MIC values of < 0.6 μg/mL for the N(2,6-diisopropylphenyl)- and N(mesityl)-substituted salts against *S. aureus* and *B. subtilis* imply that these compounds are particularly effective antimicrobials. Although the MIC values for these imidazolium salts are higher against the Gram-negative bacteria, they are still the most active of the imidazolium salts that were tested.
